# Polydopamine‐Functionalized Zinc Oxide Nanoparticles as a Root Canal Sealer: Characterization, Biological, and Physicochemical Properties

**DOI:** 10.1155/bca/7142405

**Published:** 2025-12-11

**Authors:** Arul Nayagi Raj, Aditya Shetty, Lakshmi Nidhi Rao

**Affiliations:** ^1^ Department of Conservative Dentistry and Endodontics, AB Shetty Memorial Institute of Dental Sciences, NITTE (Deemed to be University), Mangaluru, 575018, Karnataka, India, nitte.edu.in

**Keywords:** antibacterial, cytotoxicity, polydopamine, sealing ability, zinc oxide nanoparticle

## Abstract

**Background:**

Polydopamine (PDA) exhibits superior adhesion and notable bioactive characteristics, such as antimicrobial activity and favorable biocompatibility with host tissues, while zinc oxide (ZnO) nanoparticles (NPs) have proved to provide antibacterial and remineralizing properties. Combining these benefits, PDA with ZnO NP (PDA@ZnO NP) could offer superior antimicrobial activity, adhesion, and biocompatibility, possibly making it a promising alternative to conventional sealers like AH Plus (Dentsply Sirona, Germany) and zinc oxide eugenol (ZOE) (Prevest DenPro Limited, India).

**Aim:**

An effort has been made in this study to assess and compare the physicochemical properties, sealing efficiency, antibacterial potential, and biocompatibility of PDA@ZnO NP‐based root canal sealer with conventional AH Plus and ZOE sealers.

**Materials and Methods:**

The PDA@ZnO NP sealer was synthesized and characterized using scanning electron microscopy (SEM), Fourier transform infrared (FTIR) spectroscopy, and zeta potential analysis. Physicochemical properties, including setting time, flow, pH, radiopacity, sealing ability, and biological properties such as biocompatibility and antibacterial property against *Prevotella intermedia*, were assessed and compared with AH Plus and ZOE sealers. The sealer penetration was evaluated using confocal laser scanning microscopy (CLSM). A comparison among the 3 groups was conducted using ANOVA and then by Tukey’s honestly significant difference (HSD) test for post hoc multiple comparisons.

**Results:**

The PDA@ZnO NP sealer exhibited superior dentinal tubule penetration, enhanced adhesion, and strong antibacterial properties compared with AH Plus and ZOE; statistical tests showed significant differences among groups for sealing ability and antibacterial activity (*p* < 0.05). Cytotoxicity assays indicated that PDA@ZnO NP and AH Plus had comparable cytocompatibility (pairwise *p* > 0.05), while ZOE showed a transiently lower viability at 24 h (84.3%, *p* < 0.05 vs. control).

**Conclusion:**

The outcomes of this study highlighted that the PDA@ZnO NP sealer demonstrated superior sealing ability, enhanced dentinal tubule penetration, and favorable cytocompatibility, comparable to AH Plus and more favorable than ZOE at early timepoints. It also showed strong antibacterial efficacy and improved physicochemical properties. These findings suggest that PDA@ZnO NP sealer may serve as a promising alternative for clinical endodontic applications, potentially contributing to improved treatment outcomes and long‐term success in root canal therapy.

## 1. Introduction

The primary objective of root canal treatment (RCT) is to eliminate inflamed and infected pulpal tissue, promoting healing and preventing the progression of periapical disease. This is achieved through thorough chemo‐mechanical preparation and a well‐sealed final obturation of the root canal system. While obturation plays a secondary role, it is crucial in confining any residual bacteria and preventing reinfection [1–3].

Traditional root canal filling involves a combination of sealer cement and a central core material. Gutta‐percha has been used in dentistry for over a century as an obturation material. However, relying solely on gutta‐percha for obturation has been found to provide inadequate sealing, resulting in microleakage and ultimately contributing to the failure of RCT [4, 5].

Root canal sealers are essential for filling voids, enhancing adhesion, and ensuring a proper seal during obturation. When used alongside gutta‐percha, sealers not only help seal the root canal but also entomb any remaining microbes while preventing voids in the prepared canal. According to Grossman, an ideal root canal sealer should possess excellent sealing ability, dimensional stability, a slow setting time, insolubility, and biocompatibility [6, 7].

Nanotechnology has been incorporated into dentistry for various applications, including root canal sealers, tissue regeneration, drug delivery systems, and antimicrobial treatments, primarily to enhance oral health by targeting biofilms and bacteria. Nanoparticles (NPs) are ultra‐fine particles with a diameter of less than 100 nm. NPs such as AgNP, ZnO NP, CuO NP, TiO_2_ NP, Fe_3_O_4_ NP have been shown to have excellent antimicrobial properties, as they primarily exert their effects by penetrating cellular membranes and inducing cell lysis or through the reactive oxygen species (ROS) scavenging mechanism [8, 9]. The enhanced antimicrobial activity of NPs is largely attributed to their increased surface area, which allows for greater contact points and improved interactions with bacterial cells. A larger surface area facilitates the rapid and effective penetration of biofilms, which consist of bacterial communities encased in a self‐generated matrix [10].

ZnO NPs have a wide range of uses across different dental specialties, such as restorative dentistry, endodontics, and regenerative endodontics [9].

At low concentrations, ZnO NPs exhibit antibacterial properties while causing minimal harm to living cells and tissues [11]. ZnO NPs have demonstrated strong antibacterial properties along with favorable biocompatibility characteristics [4, 12]. Research indicates that ZnO NPs can minimize apical microleakage when used in root canal sealers. Additionally, due to their NP size, they exhibit enhanced penetration into dentin tubules [4, 9].

Dopamine, a molecule belonging to the catecholamine and phenethylamine families, plays a key role in adhesion and cohesion in mussels due to its strong adhesive properties, primarily attributed to catechol groups that enable bonding to various surfaces. Several studies have explored the use of dopamine‐based compounds with distinct properties for NP coating. One such compound is polydopamine (PDA), a polymer formed through the polymerization of dopamine, known for its exceptional adhesive properties even in wet conditions. As a versatile surface coating material, PDA is widely utilized in environmental, energy, and biomedical fields, making it highly applicable in dentistry for various purposes [11].

Surface modification of NPs is essential for improving their hydrophilicity, colloidal stability, biocompatibility, and capacity to bind bioactive functional groups, thereby affecting their interactions with biological systems. PDA has been extensively utilized for coating both organic and inorganic NPs. Coating NPs with PDA enhances adhesion, provides excellent biocompatibility, and imparts antimicrobial properties [13–17].

The combined effect of ZnO NP and PDA could provide superior antibacterial action against root canal pathogens, including biofilm‐forming bacteria.

Also, the incorporation of PDA with ZnO NPs can be synthesized using green chemistry approaches, making the material environmentally friendly.

The conventionally available sealers, such as AH Plus and zinc oxide eugenol (ZOE), have several disadvantages.

AH Plus, an epoxy resin‐based sealer, has limited antibacterial activity, initial cytotoxicity, a long setting time, slight shrinkage upon setting, and difficulty in removal during retreatment due to its strong adhesion to dentinal walls [18]. Similarly, ZOE sealers exhibit drawbacks such as solubility in oral fluids, delayed setting time in moist environments, potential cytotoxicity due to eugenol, brittleness over time, and interference with resin‐based adhesive restorations [5]. These limitations impact the long‐term sealing ability and biocompatibility of conventional sealers.

Considering these limitations, a novel endodontic sealer incorporating PDA@ZnO NP was developed, intended to enhance physicochemical and biological properties. This innovative composition is designed to exhibit superior antimicrobial efficacy, enhanced biocompatibility, and improved sealing ability, making it a more effective material for root canal therapy.

## 2. Materials and Methods

This study had procured institutional ethical clearance (Ref. no. ETHICS/ABSMIDS/350/2023).

### 2.1. Synthesis of PDA@ZnO NP

For the synthesis of the material, 2 g of zinc oxide (ZnO) NP along with 0.5 g of barium sulfate were crushed manually using a mortor and pestle for 15 min. The material was then collected in a crucible and kept for heating for 2 h at 100°C.

A 0.15 wt% dopamine solution is added to distilled water and sonicated (Samara Instruments, India) for 5 min.

The powder mixture is then added to the dopamine solution (Merck, Switzerland) and sonicated for another 5 min. The suspended solution is then kept under a magnetic stirrer (Lab Solution, India) for 5 h at the speed of 50 rpm. The solution was then kept for drying under vacuum for 24 h.

The powdered material containing PDA@ZnO NP was obtained [11].

### 2.2. Material Characterization

The characterization of the material was done for evaluating the surface and chemical morphology. Structure and morphology were evaluated using scanning electron microscopy (SEM) (7610FPLUS, Jeol, Japan) at 15,000x and 30,000x magnification at 3.5 kV, and energy dispersive x‐ray spectroscopy (EDS) spectrum was also performed at 3.5 kV accelerating voltage with a magnification of 5000x, using an Octane Elect Plus detector.

To examine the chemical composition of the surface‐functionalized ZnO NP, Fourier transform infrared (FTIR) spectroscopy (Bruker Tensor 27 spectrometer) was performed.

Zeta potential (ZSU1002 Low Volume Disposable Sizing Cell) was done to evaluate the surface charge and size distribution. DLS and zeta potential measurements were performed on samples dispersed in distilled water after 5 min of bath sonication; reported DLS values correspond to hydrodynamic diameter (nm) from the intensity distribution, and polydispersity index (PDI) values near 1.00 indicate broad heterogeneity and likely aggregation rather than monodispersity.

### 2.3. Setting Time

To evaluate the setting time, the sealers were mixed and placed in ring molds measuring 10 mm × 2 mm. The samples were then incubated at 37°C for 1 hour. A custom‐made Gilmore apparatus was used for setting time assessment. This device features 50 and 100 g nonsharp circular indenters, which were applied vertically onto the sealer’s surface. The test concluded once the sealer no longer retained an indentation. The initial measurement interval of 1 hour was adjusted to five‐minute intervals as the setting process progressed. The setting time was defined as the duration from the start of mixing until the sealer had fully set [5, 19, 20].

### 2.4. Flow

A graduated syringe was used to apply 0.05 mL of sealer onto a 5 mm thick glass plate.

Three minutes after mixing began, a second glass plate weighing 20 g, along with an additional 100 g weight, was placed at the center of the sealer. The maximum and minimum diameters of the sealer were measured ten minutes after the mixing process started. If the variation between the diameters was within 1 mm, the mean diameter was recorded [20].

### 2.5. pH

For the evaluation of pH, the synthesized sealer along with AH Plus (Dentsply, USA) and ZOE sealer (Prevest, India) were taken for comparison. The pH was measured using a digital pH meter (Systronics MK‐VI, India) with an accuracy of 0.01. Before each measurement, the meter was calibrated using a standard solution, and the electrode was rinsed with distilled water after every reading. The first pH measurement was recorded immediately after mixing the sealer, the second after 24 h, and the third at 120 h. For each sample, three pH readings were taken at each time interval, with a total of 15 samples analyzed, with 5 samples in each group, respectively [21] (Table 1).

**Table 1:** Results of pH.

		Duration	Mean value	Mean difference	*p* value (*p* < 0.05)
AH plus	ZOE sealer	Immediate	8.8	−1.2333	0.0934
		24 h	8.1		
		120 h	7.9		

AH plus	PDA@ ZnO NP	Immediate	8.6	0.4333	0.6584
		24 h	7.8		
		120 h	7.1		

ZOE sealer	PDA@ ZnO NP	Immediate	7.1	1.6667	0.0307
		24 h	6.6		
		120 h	6.1		

*Note:* All sealers exhibited a declining pH over the 120 h period. There is a significant difference (*p* = 0.03) between ZOE sealer and PDA@ZnO NP. There is no significant difference between AH Plus and ZOE Sealer and AH Plus and PDA@ZnO NP.

### 2.6. Radiopacity

For the evaluation of the radiopacity of dental sealers, the sealers were mixed and kept in a mold of 5 × 2 mm. Once the sealers were set, they were radiographed along with enamel and dentin. The aluminum step wedge was kept for the comparison of the radiopacity. An orthopantomography (Planmeca, Finland) was taken; a 30 cm focus‐film distance, 54 kV tube voltage, 4 mA tube current, and an exposure length of 18.7 s were the exposure parameters, and it was viewed in Planmeca Romexis software. The grayscale values of each specimen were compared with those of the aluminum step wedge, which was done using the ImageJ software.

A minimum of 3 mm of aluminum is recommended by ANSI/American Dental Association (ADA) Specification No. 57 [22].

### 2.7. Biocompatibility

Biocompatibility of the tested materials was evaluated using the MTT assay (3‐{4,5‐dimethylthiazol‐2‐yl}‐2,5‐diphenyl tetrazolium bromide; Merck, Germany), which assesses cell viability and proliferation at 24, 48, and 72 h.

Human gingival fibroblast (HGF) cells were cultured in minimum essential medium alpha (MEM‐α) (Gibco BRL, Germany) supplemented with 10% fetal bovine serum (FBS) and 1% penicillin–streptomycin (Gibco BRL, Germany) and maintained at 37°C in a humidified 5% CO_2_ incubator.

Test materials—G‐1‐AH Plus sealer, G‐2‐ZOE, and G‐3‐PDA@ZnO NP—were prepared into disc‐shaped samples (4 mm diameter × 3 mm height), allowed to set, and then UV sterilized. Material extraction was carried out by immersing the discs in complete culture medium (MEM‐*α* + 10% FBS) at a surface area‐to‐volume ratio of 3 cm^2^/mL and incubated for 24 h at 37°C. The resultant extraction medium was then collected and filtered for use in the MTT assay.

HGF cells were seeded at a density of 1 × 10^4^ cells/well in a 96‐well plate and allowed to adhere for 24 h. Subsequently, the culture medium was replaced with 100 μL of the extraction medium, which was used either undiluted or as a 1:1 dilution with fresh complete medium to ensure adequate nutrient supply and to assess potential dose‐dependent effects. Cells cultured in standard complete medium alone served as the negative control (100% viability).

After 24, 48, and 72 h of incubation with the extract, 20 μL of MTT solution (5 mg/mL) was added to each well, followed by incubation for 4 h at 37°C. The resulting purple formazan crystals were dissolved using 100 μL of DMSO, and the absorbance was measured at 570 nm using a microplate reader (Benchmark SmartReader, Sigma Aldrich).

Cell viability (%) was calculated using the formula as follows:

(1) cell viability %=A570 treated sampleA570 untreated control×100.

A570 (treated sample) = absorbance of the well containing cells treated with the material extract.

A570 (untreated control) = absorbance of the well containing cells in standard complete medium (no material exposure).

Statistical analysis was performed using SPSS software (Version 23.0). One‐way analysis of variance (ANOVA) followed by Tukey’s multiple comparison test was used to determine significant differences between groups. A *p* value < 0.05 was considered statistically significant.

### 2.8. Antibacterial Property

The antibacterial activity of the sealers was evaluated against *Prevotella intermedia*. The bacteria were cultured in preproduced, anaerobically sterilized brain–heart infusion broth (BHIb) supplemented with hemin (5 mg/L) and menadione (0.5 mg/L). For the agar diffusion test, brucella blood agar plates were used. Bacterial inoculation was performed using a sterile cotton‐tipped applicator, and four wells (3 mm in depth and 5 mm in diameter) were created in each agar plate. These wells were then filled with freshly prepared sealers. The plates were incubated in an anaerobic chamber maintained at 37°C with a gas mixture of 5% CO_2_, 10% H_2_, and 85% N_2_ for 1 week. After incubation, the diameters of the bacterial inhibition zones were measured and recorded for each sealer. A total of 12 agar plates were used per sealer. The experimental results were statistically analyzed using the Kruskal–Wallis test, with a significance level of *p* < 0.001 (Table 2).

**Table 2:** Evaluation of antimicrobial property.

Group	Mean (mm)	Standard deviation
Group 1 (PDA@ZnO NP)	6.73	0.40
Group 2 (ZOE)	3.06	0.29
Group 3 (AH Plus)	5.47	0.29

*Note:* Group 1 has the highest mean value (6.73), indicating that it exhibited the strongest antibacterial effect. Group 2 has the lowest mean (3.06) with a relatively low standard deviation (0.29), suggesting that this group had a consistently weaker antibacterial effect. Group 3 has a mean of 5.47, which is higher than Group 2 but lower than Group 1. The Kruskal–Wallis test was performed to determine whether there were statistically significant differences between the three groups *p* value: 1.66 × 10^−7^. Since the *p* value is much smaller than 0.001, it indicates a highly significant difference among the three groups.

### 2.9. Sealing Ability

Evaluation of sealing ability was conducted using a confocal scanning laser microscope. Thirty‐six extracted single‐rooted maxillary and mandibular teeth, extracted for orthodontic or periodontal reasons, were collected. Sample size was determined using nMaster software (Version 2). Teeth with extensive caries, structural anomalies, restorations, visible cracks, or fractures were excluded. Collected teeth were cleaned, disinfected, and stored following OSHA and CDC guidelines in saline. All samples were decoronated at the cementoenamel junction, and the pulp was extirpated. Working length was measured, and biomechanical preparation was done using the Universal ProTaper file system up to F3. Irrigation was performed with 3% sodium hypochlorite, followed by a final rinse with 17% EDTA and saline. Canals were dried, and samples were divided into three groups (*n* = 12 each) based on the sealer used: Group 1–PDA@ZnO NP sealer, Group 2–ZOE sealer, and Group 3–AH Plus sealer. The access cavity was sealed with GIC, and samples were stored at 37°C in a humidifier for 24 h. Afterward, two layers of nail varnish were applied, leaving the apical 2 mm exposed, and samples were submerged in Rhodamine B dye for 24 h. Sectioning was done at coronal, middle, and apical levels using a diamond disc, and samples were analyzed under a confocal scanning microscope (Zeiss LSM 880, Germany) (Figures 1, 2, and 3). Sealer penetration depth into dentinal tubules was assessed using fluorescence imaging at 10x magnification, as higher magnifications (like 20x or 40x) offer greater detail but limit the area that can be observed [23]. Measurements were recorded using digital software, and the average penetration depth was calculated. Statistical analysis was performed using ANOVA and Tukey’s honestly significant difference (HSD) test in SPSS (Version 23.0), with significance set at *p* < 0.05 (Tables 3 and 4).

Figure 1Confocal laser scanning electron microscope images of PDA@ZnO NP. (a) Coronal, green line showing maximum depth of penetration; (b) middle; (c) apical, the green line depicts the perimeter, and the violet line shows the areas of maximum sealer penetration.

**Figure:**
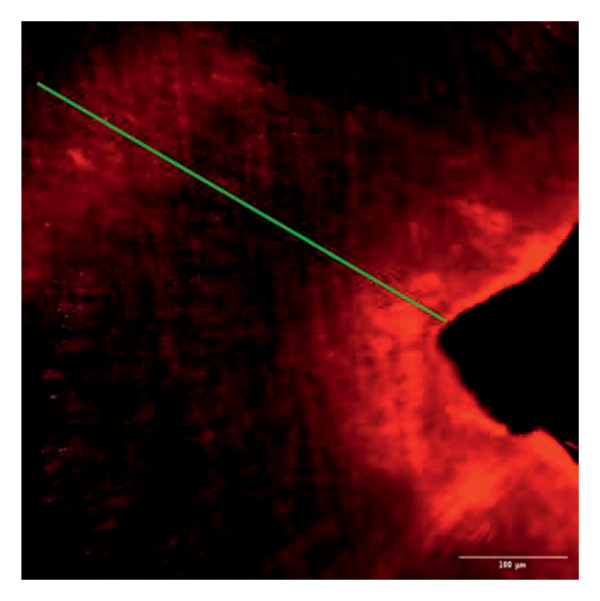
(a)

**Figure:**
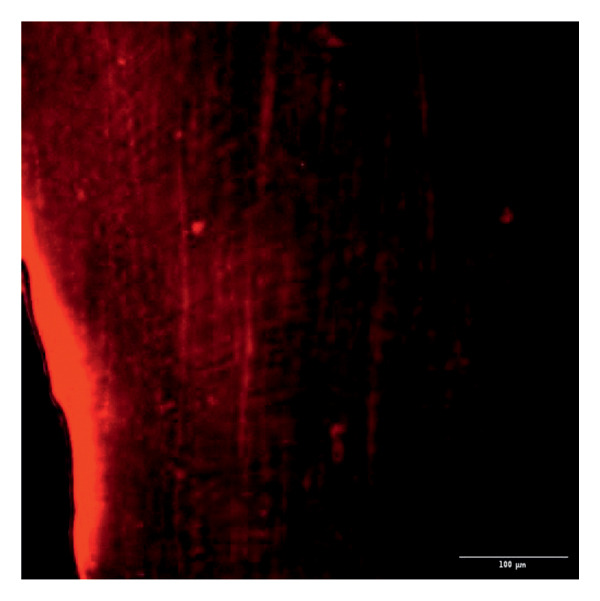
(b)

**Figure:**
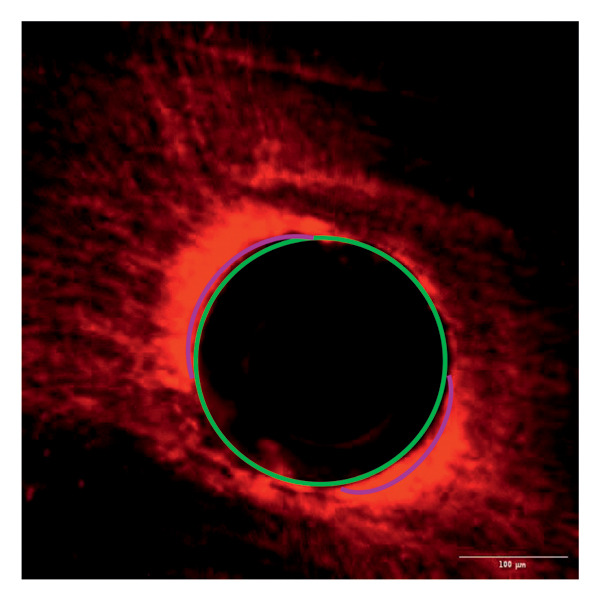
(c)

Figure 2Confocal laser scanning electron microscope images of AH Plus sealer. (a) Coronal. (b) Middle. (c) Apical.

**Figure:**
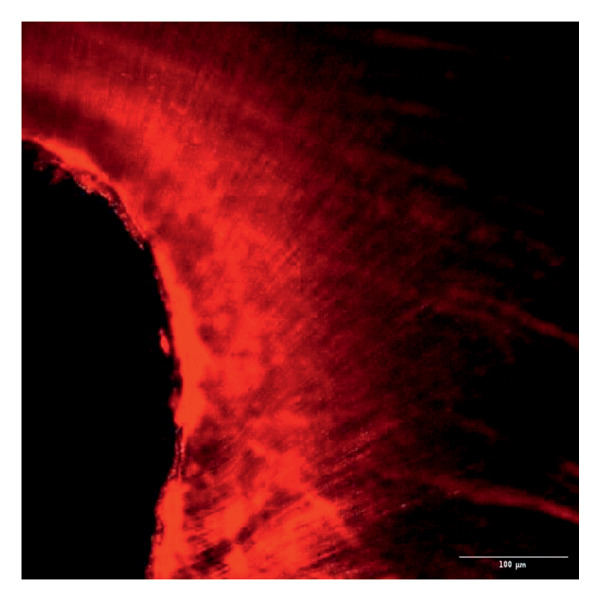
(a)

**Figure:**
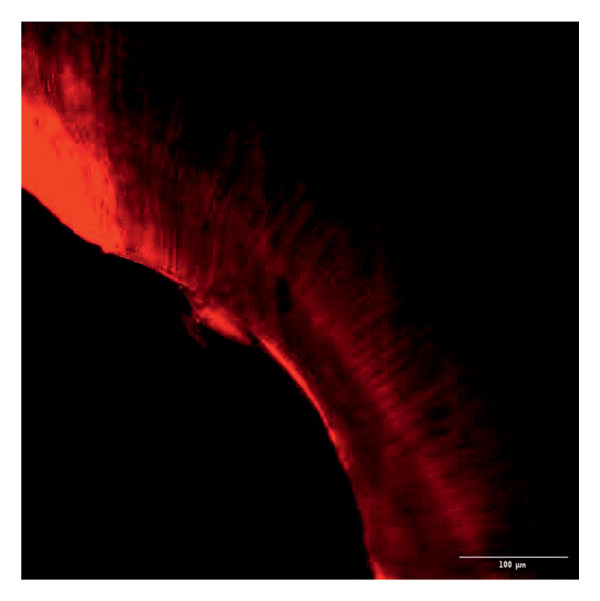
(b)

**Figure:**
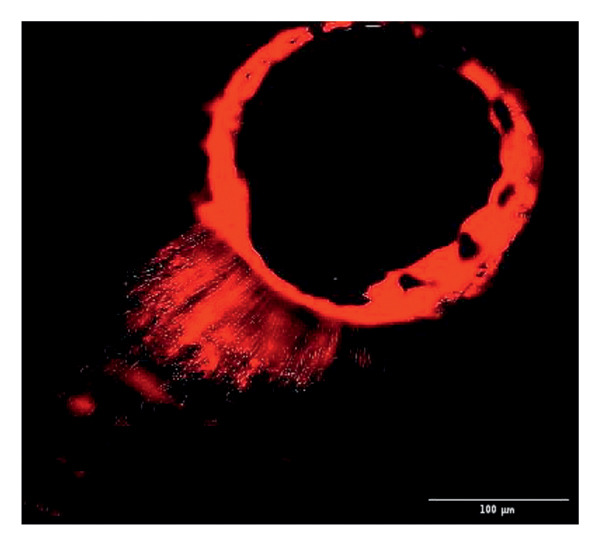
(c)

Figure 3Confocal laser scanning electron microscope images of ZOE sealer. (a) Coronal. (b) Middle. (c) Apical.

**Figure:**
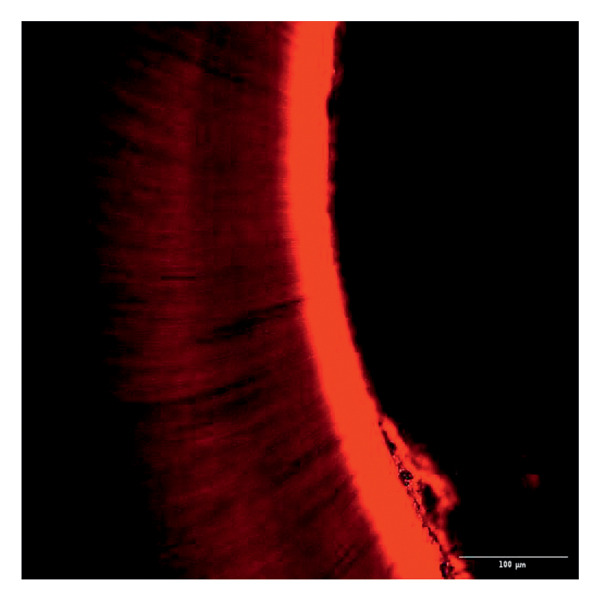
(a)

**Figure:**
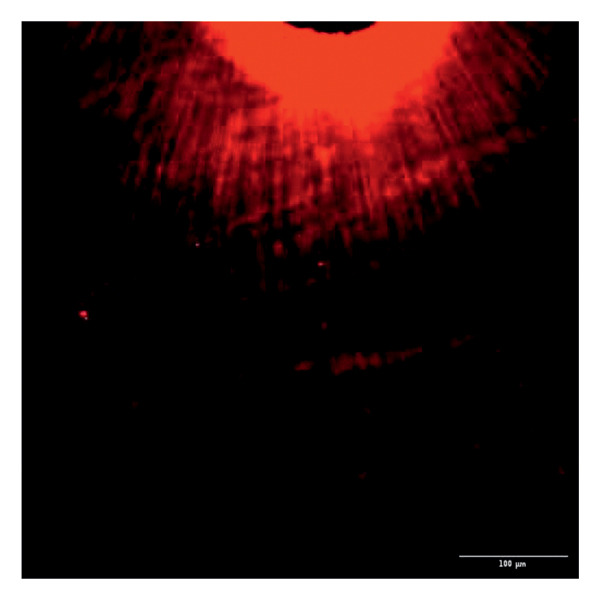
(b)

**Figure:**
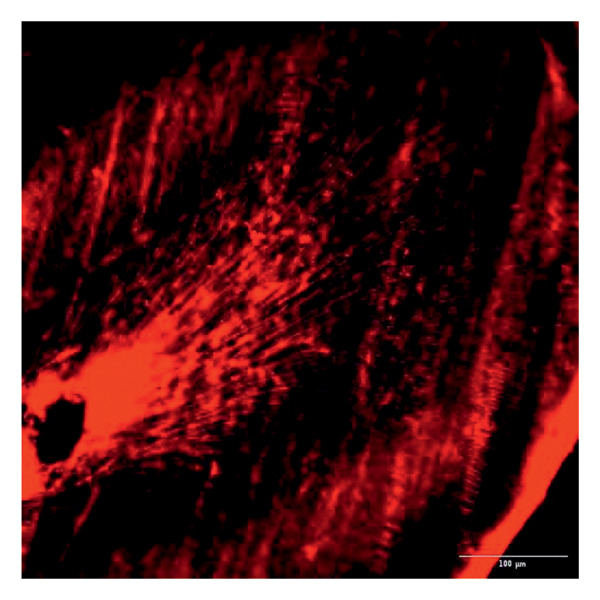
(c)

**Table 3:** Comparison of depth of penetration (μm) of the sealers in the 3 different zones.

Group	Coronal mean ± SD (μm)	Middle mean ± SD (μm)	Apical mean ± SD (μm)
Group 1 (PDA@ZnO NP)	535.92 ± 7.37	528.45 ± 13.23	463.15 ± 24.02
Group 2 (ZOE)	353.74 ± 34.39	390.35 ± 45.94	209.82 ± 13.34
Group 3 (AH plus)	464.35 ± 30.38	420.77 ± 12.72	415.92 ± 10.48

**Table 4:** Tukey’s post hoc test results.

Group 1	Group 2	Mean difference	*p* value	Significant (*p* < 0.05)
Group 1	Group 2	180.33	< 0.0001	Yes
Group 1	Group 3	36.54	< 0.0001	Yes
Group 2	Group 3	141.52	< 0.0001	Yes

## 3. Results

### 3.1. Material Characterization

**Figure 4:**
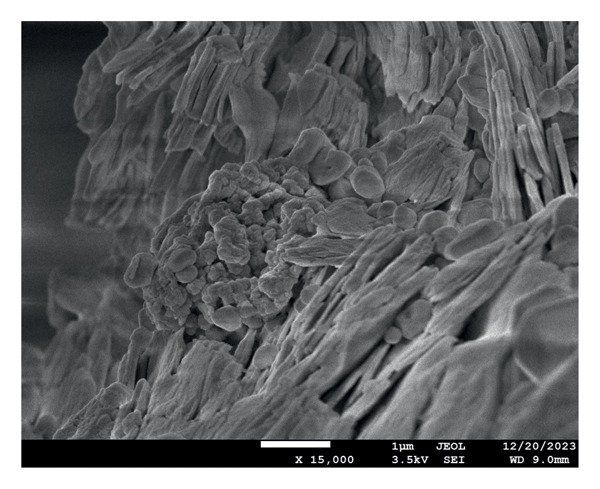
Rod‐ and needle‐shaped particles at 15,000x magnification at 3.5 kV.

**Figure 5:**
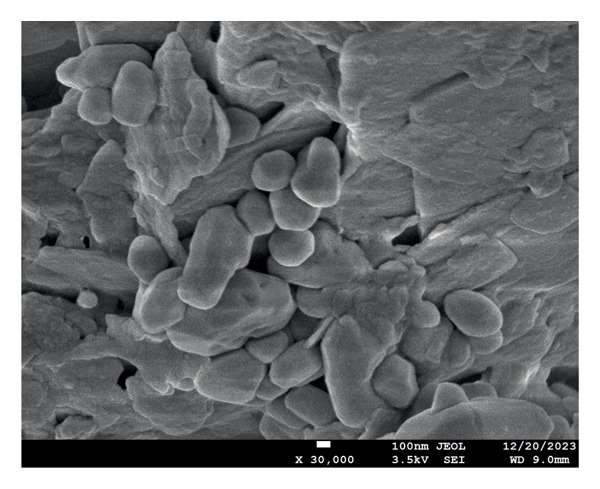
At 30,000x magnification at 3.5 kV, showing spherical‐shaped particles confirming the formation of PDA.

**Figure 6:**
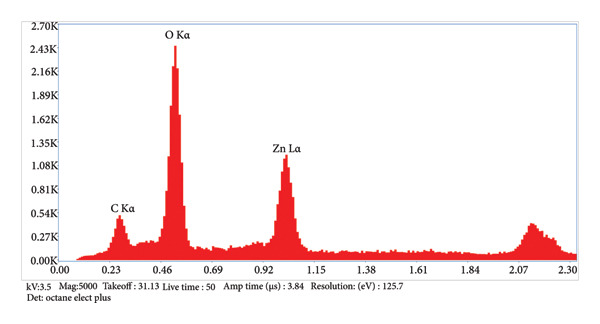
Image of EDS analysis.

**Figure 7:**
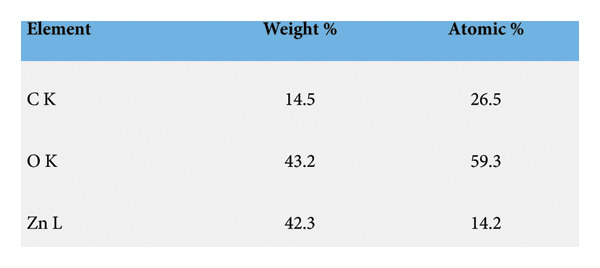
Table showing EDS analysis.

The SEM images confirm the successful formation of PDA@ZnO NPs with well‐defined morphology. Figure 4 (15,000x magnification) shows a cluster‐like arrangement with embedded NPs, indicating uniform PDA coating and strong interparticle adhesion. The observed surface roughness and granular texture suggest effective PDA deposition. Figure 5 (30,000x magnification) offers a magnified view of PDA@ZnO NP, highlighting a combination of spherical and rod‐like forms enclosed within a dense matrix, indicative of consistent PDA coverage. The lengths were about 190–500 nm. EDS showed the presence of Zn, O, and C atoms (Figures 6 and 7). The presence of 14.5% weight of C atoms confirms the formation of the PDA coating over the ZnO NP.

Figures 6 and 7 show the EDS spectrum of the sample, showing the elemental composition.The prominent peaks correspond to oxygen (O Kα), carbon (C Kα), and zinc (Zn Lα), indicating the presence of these elements in the analyzed region. The analysis was performed at 3.5 kV accelerating voltage with a magnification of 5000x, using an Octane Elect Plus detector.

**Figure 8:**
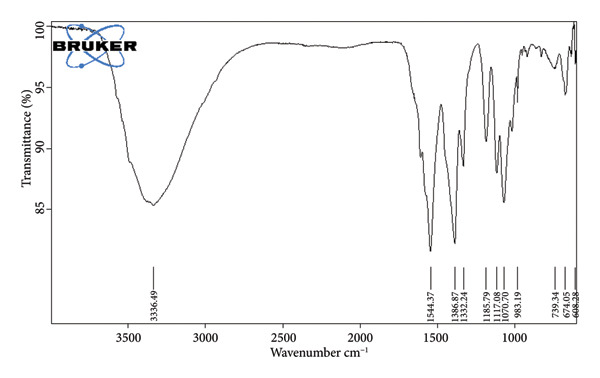
FTIR analysis.

In FTIR analysis, the peak at 3336 cm^−1^ corresponds to O‐H stretching, typically found in alcohols and phenols. The 1544.37 cm^−1^ peak indicates C=C bonds, common in alkenes and aromatics, while 1386, 983, 739, 674, and 608 cm^−1^ are associated with C‐H bending vibrations. Peaks at 1332 and 1117 cm^−1^ suggest C‐N stretching, characteristic of amines and amides. The 1185 and 1070 cm^−1^ peaks correspond to C‐O stretching, found in ethers, esters, and carboxylic acids. The spectrum confirms the presence of hydroxyl (O‐H), C=C, C‐H, C‐O, and C‐N functional groups, as seen in Figure 8.

### 3.2. Zeta Potential

**Figure 9:**
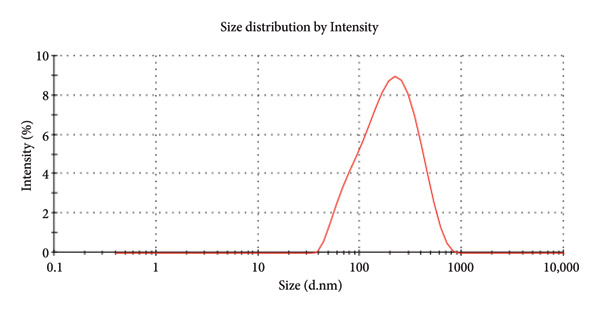
DLS intensity distribution of PDA@ZnO dispersion showing a dominant hydrodynamic diameter peak at ∼1542 nm (intensity mode); PDI = 1.00, indicating broad size aggregation.

**Figure 10:**
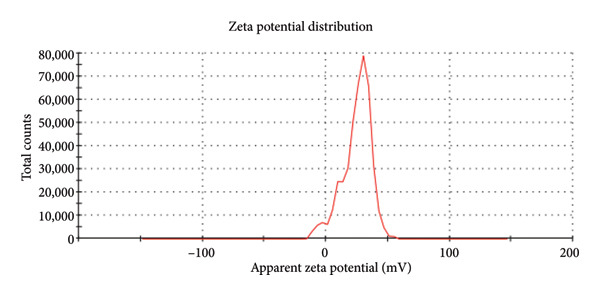
Zeta potential distribution of PDA@ZnO dispersion showing mean zeta potential −7.85 mV (zeta deviation 21.5 mV) and conductivity 1.55 mS/cm, indicating low electrostatic stabilization and propensity for aggregation.

DLS intensity distribution showed a dominant hydrodynamic diameter peak at ∼1542 nm, and the PDI was 1.00, indicating a very broad size distribution consistent with substantial aggregation (Figure 9). The measured zeta potential was −7.85 mV with a zeta deviation of 21.5 mV, consistent with limited electrostatic stabilization and a tendency toward particle clustering (Figure 10). These data indicate that the DLS‐measured hydrodynamic size reflects aggregated clusters rather than the primary NP core size observed by SEM.

### 3.3. Setting Time

According to ISO Standard 6876:2012, the setting time of a sealer should not exceed more than 10% of the time stated by the manufacturer. The PDA@ZnO NP sealer exhibited an initial setting time of 30 min and a final setting time of 150 min. The setting time for ZOE sealer is reported to be 45–60 min, while AH Plus sealer’s initial setting time is 4 h and final setting time is 8 h, according to the manufacturer’s instructions.

### 3.4. Flow

The average flow of the sealer was found to be 22.5 mm. For comparison, the flow of AH Plus sealer is 36 mm, and ZOE sealer is 30 mm. According to ISO 6876:2012 guidelines, the flow of a sealer must be at least 17 mm. The PDA@ZnO NP sealer meets these guidelines.

### 3.5. pH

A one‐way ANOVA analysis and Tukey’s HSD post hoc test were performed. All sealers exhibited a declining pH over the 120‐hr period. There is a significant difference (p = 0.03) between ZOE Sealer and PDA@ZnO NP. There is no significant difference between AH Plus and ZOE Sealer & AH Plus and PDA@ZnO NP.

### 3.6. Radiopacity

According to ISO 6876:2012, the radiopacity of the sealer must be equivalent to not less than 3 mm of aluminum. In this study the experimental sealer: 5.5 mm Al, AH Plus sealer: 5 mm Al, ZOE sealer: 6.5 mm Al, dentin: 2.5 mm Al, enamel: 3.0 mm Al.

### 3.7. Biocompatibility

One‐way ANOVA, followed by Tukey’s multiple comparison test, was used for statistical analysis. Data are presented as mean cell viability percentages ± standard error (SE), based on direct absorbance values. A *p* value < 0.05 was considered statistically significant.

**Figure 11:**
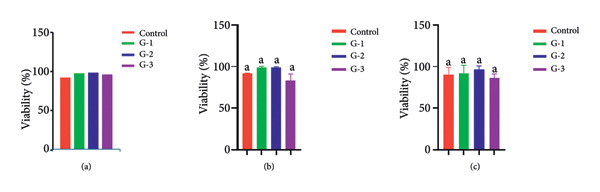
Graph showing viability of human gingival fibroblasts in all experimental groups analyzed by MTT assay. (a) Viability in 24 h; (b) viability in 48 h; (c) viability in 72 h. G‐1: AH plus; G‐2: ZOE; G‐3: PDA@ ZnO NP; control: human gingival fibroblasts.

At 24 h, the control group showed a mean cell viability of 92.5%. Group 1 (AH Plus) and Group 3 (PDA@ZnO NP) demonstrated viabilities of 91.2% and 90.8%, respectively, and were not significantly different from the control. In contrast, Group 2 (ZOE) exhibited a significantly lower viability of 84.3% (*p* < 0.05) compared to the control (Figure 11(a)). However, no significant differences were observed among the three experimental groups.

At 48 h, the control group exhibited a viability of 91.4%, while Group 1, Group 2, and Group 3 showed values of 89.7%, 87.9%, and 90.2%, respectively (Figure 11(b)). There were no statistically significant differences between the control and any of the experimental groups, nor among the groups themselves.

At 72 h, the control group showed a viability of 90.5%, with Groups 1, 2, and 3 showing values of 89.5%, 88.1%, and 91.1%, respectively (Figure 11(c)). Similar to the 48‐h results, no significant differences were observed between the control and the experimental groups or among the experimental groups.

These findings indicate that all tested materials exhibited good cytocompatibility. PDA@ZnO NP and AH Plus showed comparable viability (pairwise *p* > 0.05), while ZOE demonstrated a significant reduction at 24 h (84.3%, *p* < 0.05 vs. control); this effect diminished over time, and no significant differences were observed among groups at 48 and 72 h. Evaluation of antimicrobial property: Group 1 has the highest mean value (6.73), indicating that it exhibited the strongest antibacterial effect. Group 2 has the lowest mean (3.06) with a relatively low standard deviation (0.29), suggesting that this group had a consistently weaker antibacterial effect. Group 3 has a mean of 5.47, which is higher than Group 2 but lower than Group 1. The Kruskal–Wallis test was performed to determine whether there were statistically significant differences between the three groups–*P* value: 1.66 × 10⁻⁷. Since the *P* value is much smaller than 0.001, it indicates a highly significant difference among the three groups.

### 3.8. Sealing Ability

Group 1 (PDA–ZnO NP sealer) showed the highest mean sealer penetration, with the coronal region having the greatest values, followed by the middle and apical regions. Group 3 (AH Plus) had moderate penetration, while Group 2 (ZOE sealer) had the lowest. In Groups 1 and 3, the standard deviation of penetration depth was greater in the apical region, indicating more variability among samples at this level. Tukey’s post hoc test confirmed significant differences among all groups, with Group 1 showing the highest penetration in all three regions, followed by Group 3, and Group 2 with the least (*p* < 0.05).

## 4. Discussion

The setting time of a root canal sealer is a crucial factor influencing clinical handling and long‐term success. According to ISO 6876:2012, an ideal sealer should have a setting time that allows sufficient working time while ensuring timely hardening [16]. In this study, the experimental PDA@ZnO NP sealer exhibited a setting time of 30 min (initial) and 150 min (final), which is significantly shorter than AH Plus (4–8 h) but similar to ZOE (45–60 min). This aligns with previous studies that have shown that NP incorporation can accelerate the setting reaction due to increased surface reactivity [17, 18]. Faster setting times may enhance clinical efficiency but should be balanced with adequate flow and adaptation.

An optimal flow ensures the sealer effectively coats the canal walls and penetrates dentinal tubules. The experimental sealer demonstrated a flow of 22.5 mm, meeting ISO standards but lower than AH Plus (36 mm) and ZOE (30 mm). These results suggest that the addition of ZnO NPs increases viscosity, reducing flow compared to resin‐based sealers [6]. Studies on calcium silicate‐based sealers have also reported reduced flow when NPs are incorporated, supporting our findings [6, 18]. DLS intensity measurements showed a dominant hydrodynamic diameter at ≈1542 nm with a PDI of 1.00, indicating a very broad size distribution and substantial aggregation in the aqueous dispersion. A PDI approaching 1.00 denotes high heterogeneity and low confidence in a single‐number mean. Consequently, the DLS result should be interpreted as hydrodynamic cluster size rather than primary NP core size. The measured zeta potential (−7.85 mV, zeta deviation 21.5 mV) signals limited electrostatic stabilization and is consistent with the observed aggregation. SEM imaging revealed primary particle morphologies in the 190–500 nm range with PDA surface coating; differences between SEM and DLS arise because SEM measures dry primary particle dimensions while DLS reports hydrated, volume‐weighted hydrodynamic sizes that are heavily biased toward larger aggregates. Aggregation under the reported dispersion conditions may influence dispersion‐dependent properties such as effective surface area, ion release kinetics, and interaction with the biological environment [24, 25].

FTIR analysis confirmed the presence of functional groups indicative of PDA and ZnO integration. The peaks at 3336 cm^−1^ (O‐H stretching), 1544 cm^−1^ (C=C), and 1386 cm^−1^ (C‐H bending) suggest successful PDA coating, comparable to previous findings in biomaterials research [12, 13]. This confirms that PDA modification does not alter the core chemical structure but enhances surface characteristics.

An increase in pH is associated with enhanced antibacterial activity in endodontic sealers, particularly against *Enterococcus faecalis*, as the alkaline environment disrupts bacterial cell walls and biofilms while promoting periapical healing. In this study, however, all sealers demonstrated a gradual decline in pH values over 120 h; ZOE maintained a significantly higher alkalinity compared to PDA@ZnO NP (*p* = 0.03), likely due to the sustained hydroxyl ion release from eugenol‐based formulations, whereas PDA@ZnO NP showed a progressive reduction in pH as ZnO NPs stabilized over time. In contrast, the lower pH values observed for PDA@ZnO NP may be attributed to the gradual release and stabilization of Zn^2+^ ions and the buffering effect of the PDA coating, which likely limited hydroxyl ion accumulation. Despite not maintaining an alkaline environment, PDA@ZnO NP exhibited the strongest antibacterial activity among the tested sealers. This suggests that its antimicrobial efficacy is not solely pH‐dependent but also mediated by the sustained release of Zn^2+^ ions, generation of ROS, and the bioactive role of PDA. These mechanisms compensate for the lower pH, highlighting PDA@ZnO NP as a promising sealer with both antimicrobial potential and favorable biological interactions [8, 18, 25].

Biocompatibility is a fundamental requirement for endodontic sealers, ensuring that the material does not provoke cytotoxic, inflammatory, or mutagenic effects when in contact with periapical tissues. A biocompatible sealer should support periapical healing, minimize postoperative complications, and integrate well with surrounding tissues. The experimental PDA@ZnO NP sealer demonstrated high cytocompatibility, with mean cell viability > 90% at 24, 48, and 72 h. ZOE produced a transient reduction in viability at 24 h compared with the untreated control (84.3%, *p* < 0.05), but pairwise comparisons showed no significant difference between PDA@ZnO NP and AH Plus (*p* > 0.05), indicating comparable cytocompatibility between those two materials under the present in vitro conditions.

NPs, particularly ZnO NP, have been widely studied for their biocompatibility. ZnO exhibits antimicrobial properties while maintaining cytocompatibility at low concentrations. Studies have shown that ZnO‐based materials enhance fibroblast proliferation and modulate inflammatory responses. The addition of PDA further improves biocompatibility by promoting cell adhesion and proliferation. Research indicates that PDA coatings can promote adhesion, proliferation, and differentiation of stem cells, making them beneficial for tissue engineering applications [26].

Long‐term biocompatibility assessments, including subcutaneous implantation studies and in vivo histological evaluations, are essential for further validation. The present study suggests that PDA@ZnO NP sealers may serve as a promising alternative to conventional resin‐based and eugenol‐based sealers by offering cytocompatibility.

There has been a growing number of reports of anaerobic bacteria infecting root canals, particularly in cases of chronic infections. The antibacterial properties of root canal sealers can help manage infections, making their use highly recommended.

ZnO NPs exhibit significant antimicrobial activity due to their ability to produce ROS. The antibacterial properties of ZnO NPs are primarily due to the release of Zn^2+^ ions, which compromise bacterial membrane integrity. The large surface area of ZnO NP enhances bacterial interaction, allowing them to infiltrate cell walls, disrupt mitochondrial function, and interfere with essential cellular processes such as respiration, DNA replication, and protein synthesis. Their positive charge facilitates strong adhesion to negatively charged bacterial membranes, leading to increased permeability, intracellular leakage, and eventual cell lysis [11, 24]. PDA generates hydroxyl radicals and interacts with bacterial membranes through its catechol and amine groups, leading to cell damage and biofilm disruption. The presence of phenolic hydroxyl groups further enhances its antibacterial activity, while its role as a nanocarrier facilitates the targeted delivery of antimicrobial compounds. Additionally, PDA‐modified surfaces offer prolonged antimicrobial protection due to their chemically stable and biocompatible structure [27]. The negatively charged surface of PDA@ZnO promotes bacterial attachment, optimizing localized antimicrobial action while also reducing bacterial adhesion. The hydrophilic nature of PDA improves ZnO NP dispersion, ensuring better bacterial interaction and a controlled release of Zn^2+^ ions, which enhances antimicrobial efficacy.

The antimicrobial potency of resin‐based sealers may be linked to bisphenol A diglycidyl ether, a substance previously recognized as a genotoxic element in resin‐based compositions. Additionally, the polymerization‐driven emission of formaldehyde further contributes to the antibacterial efficacy of these sealers. The antimicrobial properties of ZOE are primarily due to the release of free eugenol from the material. As a phenolic compound, eugenol exhibits potent efficacy against microbial cells in their active vegetative state [28].

The success of endodontic treatment heavily depends on the ability of sealers to provide a hermetic seal, preventing microleakage and bacterial reinfection. The sealing ability of root canal sealers is influenced by factors such as material composition, flowability, polymerization shrinkage, and interaction with dentin [29]. NPs‐modified sealers, particularly those incorporating PDA and ZnO NP, have shown promise in enhancing dentinal tubule penetration.

Confocal laser scanning microscopy (CLSM) surpasses traditional methods like SEM and dye tests by providing depth‐resolved imaging of sealer penetration. Its fluorescence contrast enhances visualization of the sealer–dentin interface with minimal sample preparation [30].

The extent of sealer penetration and adaptation within horizontal sections can be clearly observed at low magnification, as indicated by the fluorescence of rhodamine B within the dentinal tubules [23].

This study compared the sealing ability of PDA@ZnO NP with AH Plus and ZOE sealers. CLSM revealed significantly greater dentinal tubule penetration for PDA@ZnO NP sealer compared to conventional resin‐based and eugenol‐based sealers. These findings align with recent studies emphasizing the role of NP incorporation in enhancing sealer performance (Sah S, Mangat P, Kumar A, Sah N, Shivakumar GC, Blasio MD, Cervino G, Minervini G, 2024) [31]. A critical property of an endodontic sealer is its ability to penetrate dentinal tubules, minimizing voids and reducing microleakage. The current study demonstrated that PDA@ZnO NP sealer exhibited 535.92 μm penetration in the coronal region, 528.45 μm in the middle region, and 463.15 μm in the apical region. This could be because of the superior penetration due to the smaller particle size and increased surface energy of the NPs. The interaction between sealer and dentin determines its long‐term sealing efficiency. The present study confirmed strong adhesion of PDA@ZnO NP sealer to dentinal tubules, likely due to electrostatic interactions between PDA and dentin collagen and ZnO NP reinforcement, which enhances cross‐linking with dentin structures. Zhou et al. reported that NP‐coated materials formed a more homogeneous dentin–sealer interface, reducing gaps and potential leakage pathways. Zhang et al. demonstrated that PDA‐modified hydroxyapatite fillers significantly enhance adhesive bonding and promote mineralization in dental applications. These properties may contribute to reduced microleakage, as observed in our study [15, 32]. The AH Plus sealer has demonstrated enhanced penetration into surface micro‐irregularities and lateral root canals due to its resin‐based composition, superior flowability, and extended setting time. These properties promote a strong interaction between the sealer and dentin, allowing for deeper infiltration and increased adhesion. Additionally, the cohesion among cement molecules further reinforces its bonding strength and resistance to dislodgment [33]. AH Plus sealer forms covalent bonds with root dentin by interacting with amino groups in collagen, enhancing adhesion. Its extended setting time and creep capacity enable deeper penetration into canal irregularities, improving mechanical interlock and bond stability [31]. Also, the tubular penetration of resin‐based sealers is not influenced by the hydraulic forces generated during obturation but rather occurs due to capillary action, which draws the sealer into the dentinal tubules [34].

The ZOE sealer had the least sealing ability in the 3 groups examined. This may be attributed to dimensional alterations of the material during setting.

The morphology of dentinal tubules in root dentin significantly influences the sealing ability of endodontic sealers. In the coronal region, tubules are more numerous and wider, while in the apical region, they become fewer and narrower. This variation affects how sealers interact with the dentin substrate [35]. In this study, the sealer penetration of all 3 sealers was more in the coronal, followed by the middle and apical.

These results could be due to the variation in dentin tubules in the different regions.

The removal of the smear layer, a byproduct of instrumentation, also plays a crucial role in sealer penetration. Eliminating this layer exposes the dentinal tubules, facilitating better sealer adhesion and penetration. Studies have shown that removing the smear layer improves the adaptation and binding of sealers to root canal walls [35].

Greater sealer penetration enhances root canal sealing by creating a mechanical interlock as the sealer plugs into dentinal tubules, reducing microleakage and bacterial infiltration. This improved seal is crucial for long‐term endodontic success. However, the study’s controlled laboratory setting does not fully replicate in vivo conditions. Future research should assess its in vivo performance and long‐term stability to validate clinical efficacy.

## 5. Conclusion

PDA@ZnO NP demonstrated superior sealing ability, enhanced dentinal tubule penetration, and favorable cytocompatibility, comparable to AH Plus and more favorable than ZOE at early timepoints. Additionally, the integration of ZnO NP and PDA enhanced adhesion and antimicrobial properties, which may contribute to improved long‐term treatment outcomes. The findings of this study highlight the potential of PDA@ZnO NP sealer as a promising alternative to conventional root canal sealers.

## Conflicts of Interest

The authors declare no conflicts of interest.

## Funding

This study was self‐funded.

## Data Availability

The data that support the findings of this study are available from the corresponding author upon reasonable request.
